# Sino-Cranial Aspergillosis in an ABO-Incompatible Renal Transplant Recipient Following a "March of Infections"

**DOI:** 10.7759/cureus.110331

**Published:** 2026-06-05

**Authors:** Jyoti Prakash Panda, Amit Langote, Harshad Nikte, Laxman Jessani, Sana Rahmani

**Affiliations:** 1 Microbiology, Apollo Hospitals, Navi Mumbai, IND; 2 Nephrology, Apollo Hospitals, Navi Mumbai, IND; 3 Otolaryngology, Apollo Hospitals, Navi Mumbai, IND; 4 Infectious Diseases, Apollo Hospitals, Navi Mumbai, IND

**Keywords:** abo-incompatible renal transplant, aspergillus fumigatus, drug–drug interaction, sino-cranial infection, tacrolimus, therapeutic drug monitoring, voriconazole therapy

## Abstract

We report the clinical management and pharmacologic challenges of sino‑cranial aspergillosis in a renal allograft recipient. A 60‑year‑old male patient underwent ABO‑incompatible (ABOi) kidney transplantation and, after a 15‑month “march of infections” that included multidrug‑resistant bacterial sepsis, rifamycin‑sparing antitubercular therapy, and new‑onset diabetes, presented with persistent unilateral headache. Four months earlier, functional endoscopic sinus surgery (FESS) for frontal sinusitis had yielded maxillary debris that cultured *Aspergillus fumigatus* but was interpreted as colonization. Magnetic resonance imaging demonstrated a left frontal pyocele with posterior frontal sinus table erosion and skull-base extension, and craniotomy specimens confirmed *A. fumigatus* on microscopy, histopathology, and culture. Surgical source control followed by systemic voriconazole (intravenous followed by oral), in the context of a high net state of immunosuppression, resulted in clinical improvement and radiologic resolution by 10 weeks with preserved graft function. Voriconazole therapy was complicated by a marked tacrolimus interaction that required major dose reduction and intensive therapeutic drug monitoring (TDM), with tacrolimus troughs normalizing by day 75. This case underscores that in immunosuppressed transplant recipients, sinus isolation of *Aspergillus *spp. should not be dismissed as benign colonization; even in the absence of unequivocal focal signs, it warrants timely imaging, otolaryngology reassessment, and early consideration of central nervous system (CNS)-penetrant triazole therapy with close TDM to prevent invasive CNS aspergillosis.

## Introduction

The paranasal sinuses, normally sterile or harboring sparse commensal bacteria, rely on intact mucosal defenses and competitive bacterial flora to suppress fungal colonization. *Aspergillus fumigatus*, ubiquitous in the environment, is inhaled daily without consequence in healthy individuals; disruption of local defenses by antibiotics, mucosal injury, or obstruction permits its establishment in the sinonasal cavity [1]. Fungal sinusitis spans a spectrum from benign, non-invasive forms - fungal ball and allergic fungal rhinosinusitis - to invasive subtypes defined histopathologically by hyphal penetration of submucosa, blood vessels, and bone, with potential extension to the orbit, skull base, and intracranial compartment [1,2]. This transition from colonization to invasion is fundamentally dictated by host immune status; in immunocompromised patients, mortality from acute invasive fungal rhinosinusitis remains high, often approaching 50-80%, and intracranial extension is associated with even poorer outcomes [2].

Among immunocompromised hosts, solid organ transplant (SOT) recipients are uniquely vulnerable [3]. The "net state of immunosuppression" - a framework integrating the dose and duration of immunosuppressants, comorbidities, mucosal barrier disruption, and prior infections with immunomodulating pathogens and their treatment-associated immune consequences - defines the probability of future infection at any given moment [4,5]. ABO-incompatible (ABOi) transplantation further amplifies this burden: rituximab-based B-cell depletion, plasmapheresis, high-dose induction steroids, and lifelong triple immunosuppression together carry an approximately 20% risk of serious fungal or viral infection, substantially exceeding that of standard ABO-compatible transplantation [6,7]. This baseline vulnerability compounds over time through a "march of infections" - each episode of bacterial sepsis, mycobacterial disease, or leucopenia progressively exhausts residual immune reserves and expands ecological niches for mold overgrowth, lowering the threshold at which a colonizing* Aspergillus* isolate can convert to invasive disease [4,8-10].

A critical hazard specific to sinus aspergillosis in transplant recipients is that immunosuppression blunts the cardinal symptoms of local invasion - fever, pain, and mucosal inflammation - making progressive disease clinically indistinguishable from colonization or routine chronic sinusitis [3,8]. Given the anatomical proximity of the paranasal sinuses to the orbit, cribriform plate, and frontal skull base, fungal hyphae can erode bone and enter the intracranial space before any clinical alarm is raised [2]. Consequently, a first isolate of *Aspergillus* from sinus material in a SOT recipient must never be reflexively dismissed as colonization; it represents the earliest - and most treatable - stage of a potentially fatal disease trajectory [3,9].

We report a case of an ABOi living-donor kidney transplant recipient in whom sinonasal *A. fumigatus*, initially labeled as colonization without systemic antifungal therapy, progressed to sino-cranial aspergillosis with frontal skull-base erosion and cerebral abscess, successfully managed by surgical debridement, central nervous system (CNS)-penetrant voriconazole, and careful immunosuppression modulation with serial therapeutic drug monitoring (TDM).

## Case presentation

Patient profile and immune substrate

A 60‑year‑old male with end‑stage kidney disease secondary to ischemic nephropathy and hypertension underwent an ABOi live‑donor kidney transplant from his spouse (AB‑positive to A‑positive) in October 2024 (Month 0). Pretransplant desensitization revealed low anti‑B isoagglutinin titers (IgG 1:2, IgM 1:1), and induction immunosuppression comprised basiliximab together with a single 200‑mg dose of rituximab. Maintenance immunosuppression consisted of tacrolimus (target trough 7-8 ng/mL), mycophenolate mofetil (MMF), and prednisolone, with stable initial graft function (nadir creatinine 1.15 mg/dL).

Post-transplant complications: "march of infections"

The patient's net state of immunosuppression escalated substantially over the next 15 months through sequential but unrelated infectious insults and their antimicrobial exposures. The early post-transplant period (December 2024; Month 2) was complicated by oral candidiasis managed with fluconazole, followed by a carbapenem-resistant *Klebsiella pneumoniae* urinary tract infection (CRE-UTI), which was managed with oral fosfomycin. In January 2025 (Month 3), the patient developed a prostatic abscess due to *K. pneumoniae* (carbapenem-sensitive) and hence required TURP (transurethral resection of the prostate) with deroofing and intravenous antibiotics. During this episode, the patient developed sepsis associated with leukopenia, which resolved after source control, temporary MMF withdrawal, and filgrastim support. In February 2025 (Month 4), the patient developed a persistent fever and a 3.5‑kg weight loss that did not respond to empiric antibiotics. Positron emission tomography-computed tomography (PET‑CT) showed hypermetabolic omental thickening, and laparoscopic biopsy confirmed granulomatous tuberculosis on histopathology, with Xpert MTB/RIF® negative on both tissue and ascitic fluid. To minimize calcineurin inhibitor interactions and avoid CYP3A4 induction, a rifamycin-free regimen of isoniazid 300 mg, ethambutol 1000 mg, and levofloxacin 500 mg twice daily was initiated with pyridoxine supplementation.

At Month 7, the patient developed new-onset diabetes after transplant (NODAT) with an HbA1c of 13.8%, which was managed with insulin (Figure 1).

**Figure 1:**
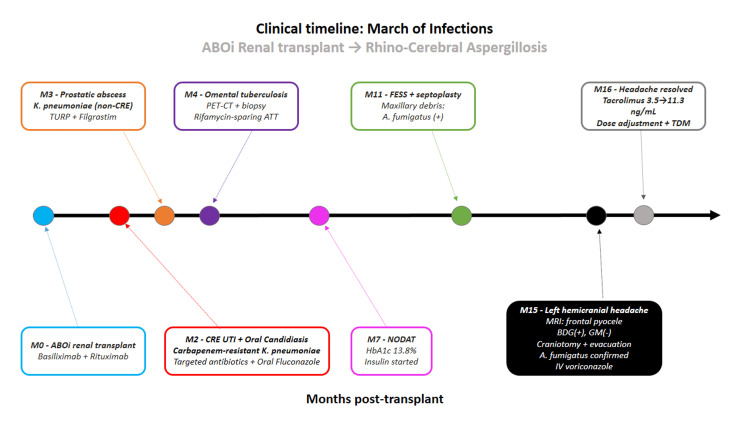
Clinical timeline illustrating the "march of infections" in an ABO-incompatible kidney transplant recipient. Key post-transplant events are shown by month: M0, ABO-incompatible renal transplant with basiliximab and rituximab induction; M2, carbapenem-resistant *Klebsiella pneumoniae* urinary tract infection and oral candidiasis treated with targeted antibiotics and oral fluconazole; M3, prostatic abscess due to* K. pneumoniae* (non-CRE) requiring TURP, intravenous antibiotics, and filgrastim-supported sepsis management; M4, omental tuberculosis diagnosed on PET-CT and biopsy and treated with a rifamycin-sparing regimen (isoniazid, ethambutol, and levofloxacin); M7, new-onset diabetes after transplant (NODAT; HbA1c 13.8%) requiring insulin; M11, functional endoscopic sinus surgery (FESS) with septoplasty and isolation of *Aspergillus fumigatus* from maxillary sinus debris; M15, progressive left hemicranial headache with MRI-defined frontal pyocele, β-D-glucan positivity and galactomannan negativity, and craniotomy confirming sino-cranial aspergillosis; and M16, headache resolution with tacrolimus trough elevation (3.5→11.3 ng/mL) prompting dose adjustment and therapeutic drug monitoring (TDM).

Sinonasal event and missed therapeutic opportunity (Months 11-15)

A deviated nasal septum with bilateral frontal sinusitis was diagnosed clinically in mid-2025, for which functional endoscopic sinus surgery (FESS) with septoplasty was performed in September 2025 (Month 11). Intraoperative findings included bilateral blocked frontal recesses and inspissated debris in the right maxillary sinus. Debris was sent for culture and sensitivity, and *A. fumigatus *was subsequently isolated from this sinus material. In the absence of systemic symptoms, the isolate was interpreted as colonization, and no systemic azole therapy was initiated at this stage.

Over the following four months, the patient experienced a progressive, left-sided periorbital and hemifacial headache with gradual worsening. This headache was dull with no associated fever or nasal discharge. This was attributed to raised intraocular pressure from bilateral immature cataracts - possibly due to NODAT and steroid use - that had been detected in the same period. Left eye cataract surgery was performed in November 2025 (Month 13); however, blurred vision and headaches persisted postoperatively. In retrospect, the headache was likely an early manifestation of contiguous orbital and intracranial *Aspergillus *extension rather than a primary ophthalmic condition.

Acute neurological presentation (Month 15)

By late January 2026 (Month 15), the headache had become severe, analgesic-refractory, and was accompanied by three to four daily vomiting episodes. An endoscopic skull‑base biopsy with left frontal pyocele drainage provided transient relief, but the headache recurred within 48 hours, prompting an urgent MRI of the brain. MRI of the brain with contrast demonstrated erosion of the posterior table of the frontal sinus, cerebritis, and meningeal enhancement, consistent with contiguous intracranial spread, culminating in a left frontal lobe skull-base abscess (Figure 2). Serum β‑D‑glucan (BDG) was positive (145.2 pg/mL), while serum galactomannan (GM) was negative (OD Index 0.1574). The total leukocyte count was 8.74 × 10⁹/L and creatinine 1.22 mg/dL without a systemic inflammatory response.

**Figure 2:**
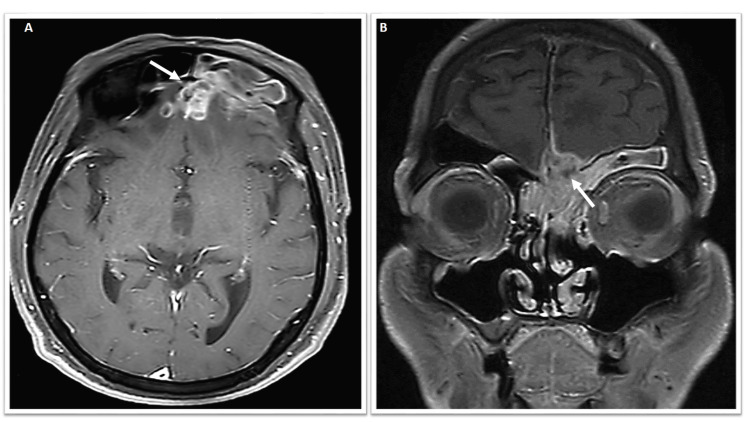
MRI brain demonstrating sino‑cranial aspergillosis. (A) Axial post‑contrast T1‑weighted image showing enhancing soft tissue at the left frontal sinus and skull base with erosion of the posterior table (arrow). (B) Coronal post‑contrast T1‑weighted image demonstrating contiguous extension from the frontal sinus across the skull base towards the left frontal lobe (arrow), consistent with a frontal pyocele and skull‑base abscess.

Management

The patient underwent a left frontal craniotomy with complete evacuation of the abscess and excision of the infected skull-base bone in late January 2026. Intraoperatively, caseous purulent material was encountered epidurally at the skull base with surrounding polypoidal mucosa; the dura was found to be breached and was subsequently repaired primarily.

Microbiological analysis of the operative specimen provided definitive diagnostic confirmation. KOH mount demonstrated septate fungal filaments, and Gram’s staining revealed abundant septate hyphae with characteristic acute-angle dichotomous branching. Culture on both blood agar and Sabouraud's dextrose agar (SDA) yielded growth of* A. fumigatus* (Figure 3), with broth microdilution (BMD) susceptibility testing confirming sensitivity to voriconazole. Bacterial cultures, mycobacterial cultures, Xpert MTB/RIF®, and *Nocardia* polymerase chain reaction (PCR) were all negative, effectively excluding co-infection. Histopathological examination confirmed the diagnosis; however, no evidence of angioinvasion was identified. According to the 2020 EORTC/MSG (European Organisation for Research and Treatment of Cancer/Mycoses Study Group) criteria, this case fulfills the definition of a proven case of invasive CNS aspergillosis (culture and histopathology proven from a sterile site) [11].

**Figure 3:**
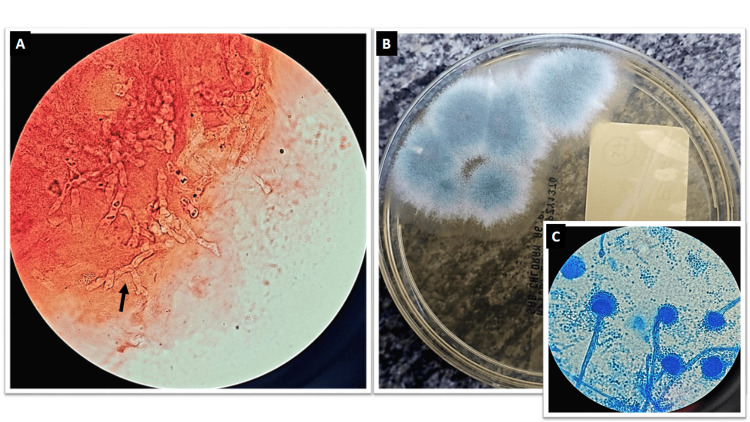
Microbiological confirmation of invasive A. fumigatus. (A) Gram-stained microscopy (1000×) of skull‑base pus shows septate hyphae with acute‑angle dichotomous branching (arrow). (B) Culture from the operative specimen on Sabouraud's dextrose agar (SDA) showing characteristic blue-green, powdery colonies of *A. fumigatus*. (C) Lactophenol cotton blue mount demonstrating typical conidial heads with columnar conidia arising from vesicles at the tips of conidiophores, confirming *A. fumigatus.*

Antifungal therapy was initiated with intravenous voriconazole (loading dose 400 mg, followed by 300 mg IV twice daily), selected for its proven CNS penetration and established efficacy in invasive aspergillosis. Immunosuppression was modulated by discontinuing MMF while continuing low-dose tacrolimus and prednisolone to balance the risk of graft rejection against the imperative of restoring immune competence. Tight glycemic control was maintained throughout with insulin therapy. Creatinine was closely monitored; however, rejection surveillance protocol with protocol biopsy was not performed.

The patient responded favorably, with progressive resolution of headache and neurological symptoms, and was discharged on oral voriconazole 200 mg twice daily with stable allograft function (serum creatinine 1.0 mg/dL) and a therapeutic voriconazole trough of 2.67 mg/L (reference range approximately 1-6 mg/L) measured at the end of Week 1.

Post-discharge follow-up and outcome

At the one‑month follow‑up (Month 16 post‑transplant/Week 4 post‑craniotomy), left hemicranial headache had markedly reduced, and neurological and otolaryngological examinations were unremarkable. Renal allograft function was stable (creatinine 0.99 mg/dL). Tacrolimus troughs had risen from 3.5 to 11.3 ng/mL after starting voriconazole, necessitating dose reduction and close TDM. Distal symmetric lower‑limb paresthesia had developed. In view of neuropathy, isoniazid and levofloxacin were replaced with a brief rifabutin-ethambutol tail (seven additional days) to complete just over 12 months of antitubercular therapy, and gabapentin was initiated for symptomatic relief. At six weeks, the headache had fully resolved, and tacrolimus troughs had partially corrected to 9.08 ng/mL, and abdominal ultrasonography confirmed resolution of omental thickening. At 10 weeks, C‑reactive protein was 3.88 mg/L, and tacrolimus troughs had normalized to 4.61 ng/mL, completing the TDM arc (3.5 → 11.3 → 9.08 → 4.61 ng/mL) (Figure 4). An MRI of the brain confirmed resolution of frontal cerebritis with only residual changes, and voriconazole 200 mg twice daily was continued to Day 90 as planned.

**Figure 4:**
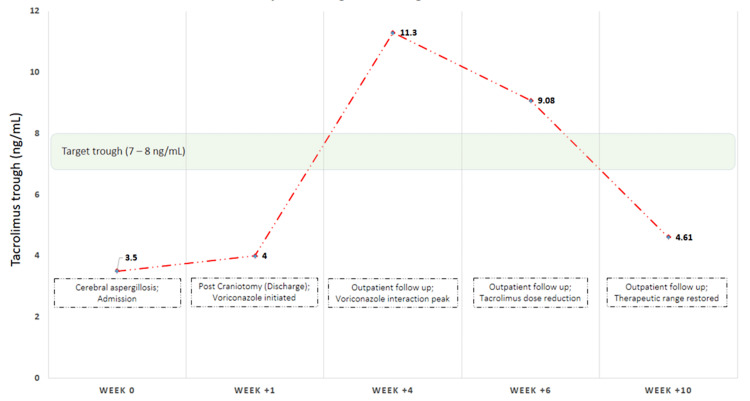
Tacrolimus therapeutic drug monitoring (TDM) around voriconazole initiation. Serial tacrolimus trough concentrations (ng/mL) are shown from Week 0 (admission with cerebral aspergillosis) through Week 10 after voriconazole initiation. Baseline troughs of 3.5 ng/mL at admission and 4.0 ng/mL at post‑craniotomy discharge rose to a peak of 11.3 ng/mL by Week 4, consistent with voriconazole‑mediated CYP3A4 inhibition. Following a 67% tacrolimus dose reduction and close TDM, troughs declined to 9.08 ng/mL in Week 6 and normalized to 4.61 ng/mL by Week 10, with graft function remaining stable. The shaded band (7–8 ng/mL) indicates the target tacrolimus trough range.

## Discussion

The march of infections

This case exemplifies the transplant infectious diseases principle of the "net state of immunosuppression" [4,5]. We use the term "march of infections" to describe the sequential accumulation of opportunistic and nosocomial infections in this patient. Although these infections were independently acquired rather than causally related, each episode and its treatment - antifungals for candidiasis, broad-spectrum antibiotics, antitubercular agents, filgrastim, and mandatory MMF withdrawal - cumulatively eroded residual immune reserves and expanded ecological niches for mold overgrowth, lowering the threshold at which a colonizing *Aspergillus* converted to invasive disease [8,10].

The portal of entry and diagnostic delay

The Month-11 sinus culture yielding *A. fumigatus* represented the earliest actionable warning and likely the portal of entry for subsequent contiguous intracranial spread via erosion of the posterior frontal sinus table. The headache was initially attributed to cataracts, which was plausible in a steroid-treated transplant recipient with poor glycemic control, but its persistence after cataract surgery was the key red flag. Systemic antifungal therapy was deferred because the *A. fumigatus* isolate from maxillary debris was interpreted as probable colonization, and there was concern about azole-calcineurin inhibitor interactions. In retrospect, earlier systemic therapy and/or expedited neuroimaging would have been reasonable given the patient’s immunocompromised state and progressive focal headache. Pragmatically, sinus isolation of *Aspergillus *spp. in an immunosuppressed host, especially with a unilateral or persistent focal headache, supports a low threshold for repeat imaging, otolaryngology reassessment, and initiating a CNS‑penetrant triazole with TDM rather than accepting “colonization” as a benign explanation [1,2,9].

Biomarker discordance: GM vs. BDG

The serum GM‑negative/BDG‑positive pattern may reflect localized, encapsulated CNS aspergillosis without angioinvasion, where limited antigen release from a walled‑off abscess reduces circulating GM detection [12]. Cerebrospinal fluid (CSF) GM or BDG might have provided supportive evidence but were not obtained [12-14]. Although false‑positive BDG results (hemodialysis, mucositis, certain antibiotics) must always be considered [15], in this context, BDG functioned as a useful serological alert, with definitive diagnosis secured by microscopy, histopathology, and culture.

Management strategy: surgery, voriconazole, and drug interactions

Management followed a combined modality approach consistent with established guidelines [9]. Craniotomy and abscess evacuation provided rapid source control and simultaneously yielded tissue for definitive microbiological diagnosis. Voriconazole was selected as first-line therapy per Infectious Diseases Society of America (IDSA) guidelines for CNS aspergillosis, given its reliable CNS penetration and proven efficacy; amphotericin B remains appropriate when azoles are contraindicated or not tolerated [9]. Voriconazole was continued for 90 days despite clinical and radiological resolution by approximately 10 weeks, because the patient remained significantly immunosuppressed; this duration was individualized in accordance with IDSA guidance to continue therapy until adequate clinical and radiological response and host immune recovery were achieved [9].

Pharmacotherapy was complicated by significant drug-drug interactions. Voriconazole, a potent CYP3A4 inhibitor, markedly increased tacrolimus exposure: within 16 days, tacrolimus troughs rose from 3.5 to 11.3 ng/mL, necessitating a 67% dose reduction (1.5 mg to 0.5 mg twice daily) and intensive serial monitoring [16,17]. Troughs subsequently corrected to 9.08 ng/mL at six weeks and 4.61 ng/mL at 10 weeks, with stable allograft function throughout. A single voriconazole trough measured on day 6 by mass spectrometry, before any rifamycin exposure, was 2.67 mg/L (therapeutic reference range approximately 1-6 mg/L), confirming adequate CNS‑penetrant drug exposure on standard oral dosing [18]; the absence of serial voriconazole levels remains a limitation, but sustained clinical and radiological response suggests adequate therapeutic exposure.

Additional complexity arose from the concurrent antitubercular regimen. To avoid CYP3A4 induction and tacrolimus destabilization, the patient received a rifamycin‑free regimen (isoniazid, ethambutol, and levofloxacin) for roughly 12 months. Near the end of this course, he developed distal symmetric lower‑limb paresthesia, attributed to multifactorial neurotoxicity: isoniazid‑related pyridoxine‑dependent neuropathy, levofloxacin‑associated axonal injury, voriconazole exposure, and pre‑existing diabetic neuropathy acting synergistically. Despite concurrent pyridoxine supplementation, isoniazid and levofloxacin were discontinued; a brief rifabutin-ethambutol tail was used to complete just over 12 months of therapy. Rifabutin, therefore, overlapped with voriconazole only transiently in this patient: the theoretical bidirectional CYP3A4 interaction (rifabutin inducing voriconazole clearance while voriconazole inhibits rifabutin metabolism and increases its toxicity risk) remains important for clinicians to recognize, but its clinical impact here was likely limited by the very short co‑administration period.

Limitations

The key limitations of this report must be noted. First, CSF GM, an established adjunctive biomarker for CNS aspergillosis, was not measured, which could have provided valuable diagnostic support [13]. Second, a protocol renal allograft biopsy was not performed during immunosuppression modulation; thus, subclinical rejection cannot be definitively excluded despite stable graft function. Finally, TDM was incomplete; while a single first-week voriconazole trough was within target, serial levels were omitted due to financial constraints, leaving the true drug exposure profile unclear.

## Conclusions

In immunosuppressed patients, isolation of *Aspergillus *spp. from sinus cultures should never be dismissed as colonization, regardless of the presence or absence of focal symptoms, and should prompt urgent imaging, ENT reassessment, and a low threshold for systemic CNS-penetrant triazole therapy with TDM. This case illustrates that delayed recognition of sinonasal *Aspergillus* in heavily immunosuppressed individuals can lead to sino-cranial aspergillosis; however, with timely neurosurgical source control and voriconazole with serial TDM-guided management of both antifungal exposure and calcineurin inhibitor levels, complete neurological recovery and allograft preservation are achievable, as defined here by complete clinical recovery, MRI normalization, and no evidence of recurrence at six-month follow-up. Furthermore, when antifungal therapy is co-administered with complex antimicrobial regimens, including antitubercular agents with CYP3A4-modulating potential, bidirectional drug interactions must be anticipated, monitored, and explicitly addressed in clinical management.
